# Deficient O-GlcNAc Glycosylation Impairs Regulatory T Cell Differentiation and Notch Signaling in Autoimmune Hepatitis

**DOI:** 10.3389/fimmu.2018.02089

**Published:** 2018-10-09

**Authors:** Xiaohua Hao, Yufeng Li, Jianwen Wang, Jiali Ma, Shuli Zhao, Xiaohui Ye, Lingling He, Junru Yang, Meixin Gao, Fan Xiao, Hongshan Wei

**Affiliations:** ^1^Beijing Ditan Hospital, Capital Medical University, Beijing, China; ^2^Central Laboratory of Nanjing First hospital, Nanjing Medical University, Nanjing, China

**Keywords:** O-GlcNAc glycosylation, autoimmune hepatitis, treg cells, EOGT, notch signaling pathway

## Abstract

Post-translational modifications such as glycosylation play an important role in the functions of homeostatic proteins, and are critical driving factors of several diseases; however, the role of glycosylation in autoimmune hepatitis is poorly understood. Here, we established an O-GlcNAc glycosylation-deficient rat model by knocking out the *Eogt* gene by TALEN-mediated gene targeting. O-GlcNAc glycosylation deficiency overtly aggravated liver injury in concanavalin-A induced autoimmune hepatitis, and delayed self-recovery of the liver. Furthermore, flow cytometry analysis revealed increased CD4^+^ T cell infiltration in the liver of rats with O-GlcNAc glycosylation deficiency, and normal differentiation of regulatory T cells (Tregs) in the liver to inhibit T cell infiltration could not be activated. Moreover, *in vitro* experiments showed that O-GlcNAc glycosylation deficiency impaired Treg differentiation to inhibit the Notch signaling pathway in CD4^+^ T cells. These finding indicate that O-GlcNAc glycosylation plays a critical role in the activation of Notch signaling, which could promote Treg differentiation in the liver to inhibit T cell infiltration and control disease development in autoimmune hepatitis. Therefore, this study reveals a regulatory role for glycosylation in the pathogenesis of autoimmune hepatitis, and highlights glycosylation as a potential treatment target.

## Introduction

Autoimmune hepatitis (AIH) is a typical immune-mediated liver disease characterized by hepatocellular inflammation and immune-mediated destruction of the hepatic parenchyma, resulting in liver failure, cirrhosis and death (1). Although understanding of the specific triggers initiating the series of events in AIH development and progression is still at its rudimentary stage, multiple clinical and basic science studies have suggested that T lymphocytes likely act as primary drivers of autoimmune responses via the innate and adaptive immune systems (2, 3). Indeed, during the development of AIH, the main population of infiltrated immune cells is largely composed of CD4^+^ and CD8^+^ T lymphocytes, whose intrahepatic accumulation is associated with increased histological severity of hepatitis (4).

The liver is often considered a unique site of immune tolerance (5), and functions to systemically inhibit autoimmune responses against ectopic antigens (6). Although the specific mechanisms underlying immune tolerance in the liver remain unclear, regulatory T cells (Tregs) are known to mediate hepatic immune tolerance (6). Tregs are central to the regulation of self-tolerance and maintenance of tissue homeostasis by preventing the activation and expansion of auto-reactive T lymphocytes that contribute to autoimmune diseases, and limiting immune responses in allergic diseases, infections, transplantation, graft-vs.-host disease, and cancer (7–9). Experimental evidence suggests that AIH with immunoregulatory dysfunction is characterized by decreased amounts of Tregs and FOXP3 expression levels (10, 11). Meanwhile, reduction of Treg amounts has been described in peripheral blood with a parallel increase of Treg frequency along with effector cell numbers in the inflamed liver tissue (12). Therefore, regulating Treg activation and expansion appears to be essential for self-systematic inhibition of AIH.

The development and maintenance of immunosuppressive function in Tregs are crucially controlled by FOXP3 expression; therefore, such regulation mainly aim to modulate FOXP3 protein expression (13). The transcriptional activity of FOXP3 is modulated by accessory extracellular signal activation and intracellular transcription factors (14–16), whose functions are precisely modulated by post-translational modifications (PTMs) (17). Various studies have demonstrated that FOXP3 is regulated by PTMs, including acetylation, ubiquitination and phosphorylation (17). Surface glycosylation is another PTM, which is ubiquitous in mammalian cells; it also regulates T cell development, trafficking and function (18). Recently, Cabral et al. (19) reported that surface glycosylation of Tregs is important in determining the Treg phenotype and suppressive potency. However, the role of glycosylation in modulating Treg development and activation in AIH is poorly understood.

Concanavalin A (Con A) is a plant lectin that is widely used for inducing acute immune-mediated hepatitis with an activated inflammatory response (20, 21). Indeed, Con A-induced AIH is a typical and well-established model for investigating T cell-mediated liver injury, closely mimicking the pathogenic mechanisms and pathological changes observed in AIH patients (22). In this study, we established a Con A-induced AIH rat model in which the *Eogt* gene was knocked out by the transcription activator-like effector nuclease (TALEN) technology. *Eogt* encodes a key enzyme for O-GlcNAc glycosylation and catalyzes the transfer of N-acetyl glucosamine to serine or threonine residues of target extracellular proteins (23). This knockout resulted in O-GlcNAc glycosylation deficiency, and was used to examine the effects of glycosylation on Treg activation and development, as well as the associated liver injury in AIH and underlying mechanisms.

## Materials and methods

### TALEN construction

**Figure 1:**
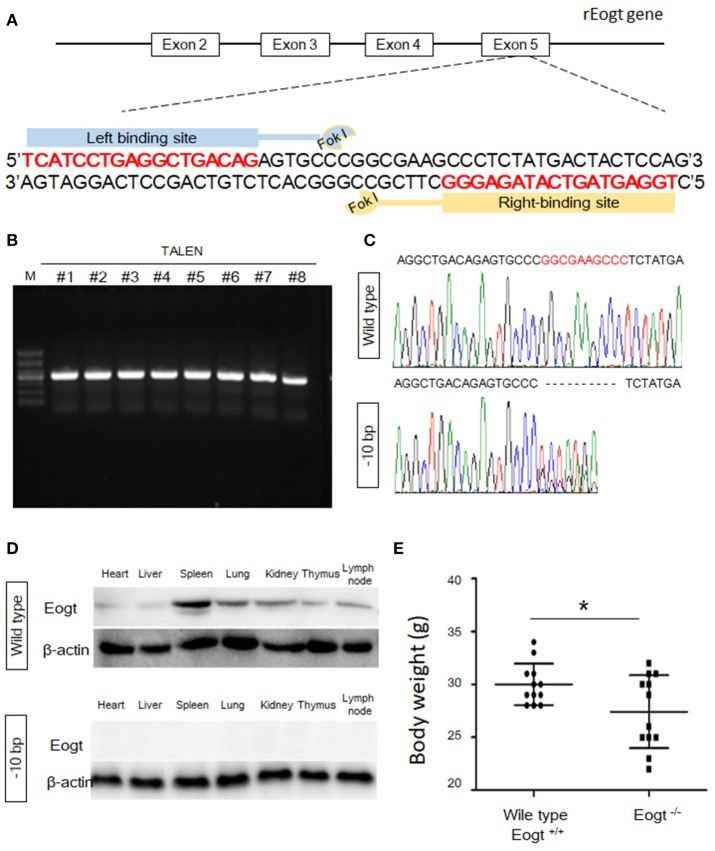
Generation of Eogt1 knockout rats by TALEN-mediated gene targeting. **(A)** Schematic representation of the rEogt1 locus and rEogt1-TALEN design. Double-stranded DNA sequence of the rEogt1 locus that was targeted with TALENs. The TALEN binding sites are marked with red; **(B)** Agarose gel electrophoresis demonstrating products of the predicted size for the rEogt1 locus in 8 healthy offspring; **(C)** Representative genomic sequencing results of rEogt mutation around the target site. The black dotted line represents nucleotide deletions; **(D)** Western blot analysis of Eogt expression in the heart, liver, spleen, lung, kidney, thymus and lymph nodes of wild type and Eogt knockout rats; **(E)** Body weights in wild type and Eogt knockout rats on day 20 after birth. ^*^p < 0.05, vs. WT control group.

A pair of TALENs targeting exon 5 of the rat *Eogt* gene (GenBank accession number: NM_001009502.1) were created by Cyagen Biosciences Inc. Each TALEN binds to 18 bp of DNA, and binding sites are separated by a 14-bp spacer region as illustrated in Figure 1A. The TALENs were assembled using TALE Toolkit (Addgene, catalog # 1000000019) according to published protocols (24). Final constructs were produced in the pRP[TALEN]-Hygro-CMV backbone plasmid (Cyagen Biosciences Inc.).

The TALEN plasmids were linearized with SmaI and used as templates for *in vitro* transcription with mMessage mMachine T7 Ultra Kit (Ambion) according to the manufacturer's instructions. Capped, polyA-tailed mRNAs were cleaned up with a MEGAclear kit (Ambion). The mRNAs were precipitated, washed and resuspended at 1 μg/μL in DEPC-treated H_2_O. TALEN mRNAs were subsequently diluted in 0.1 × TE buffer at a final concentration of 10 ng/μL, aliquoted, and stored at −80°C until use for embryo injection.

### Microinjection of TALENs in fertilized eggs

All animal-based experimental procedures were approved by the Institutional Animal Care and Use Committee, Peking University Health Science Center (SCXK: 2011-0012). Rats were bred and maintained in accordance with the Peking University Health Science Center guidelines for use of Laboratory Animals. Sprague Dawley (SD) rats (Charles River Laboratories) were housed under specific pathogen-free conditions under a 12/12 h light/dark cycle (7:00–19:00). Female embryo donors were superovulated with 25 IU of pregnant mare serum gonadotropin (Sigma) between 11:00 and 12:00, followed by administration of 25 IU of human chorionic gonadotropin (Sigma) 24 h later, and subsequently individually caged with a male stud rat. The following morning, donors were sacrificed, and embryos were collected from oviducts and cultured in M16 medium (Millipore) at 37°C in 5% CO_2_/95% air. Fertilized one-cell embryos were transferred to M2 medium (Millipore) for microinjection. TALEN mRNAs were injected into the cytoplasm using glass injection pipettes. Embryos that survived the injection procedure were surgically transferred to the oviduct of day-0.5 post coitum pseudopregnant recipient SD females that had successfully mated with vasectomized males.

### Mutation analysis

Offspring from injected embryos were screened for mutations in the *Eogt* locus by polymerase chain reaction (PCR) followed by DNA sequencing. Briefly, DNA was prepared from tail snips (~0.5 cm) using E.Z.N.A.® Forensic DNA Extraction Kit (Omega BioTek, USA) according to the manufacturer's instructions. A portion of the *Eogt* locus that overlaps with the TALEN spacer region was amplified by PCR with the forward primer 5′-GTTTGCCACCAGTCCTGTCTGAAG-3′ and reverse primer 5′-CGCTACCTTATACGGACAGTGGGA-3′. PCR reactions included Taq 2X Master Mix (New England Biolabs Inc., Ipswich, MA, USA), and the amplification program consisted of 95°C for 5 min, followed by 30 cycles of 95°C for 30 s 58°C for 30 s and 72°C for 30 s, with a final extension at 72°C for 5 min. Ten microliters of PCR products were analyzed on ethidium bromide-stained 1.5% agarose gels in Tris–borate–EDTA buffer.

In addition, *Eogt* PCR products from the founder rats were sequenced using the same primers described above. Positive founders were bred to the next generation, and the offspring were genotyped by PCR and DNA sequencing as described above.

### Con A-induced autoimmune liver injury model

Wild type (*Eogt*^+/+^) and Eogt knockout (*Eogt*^−/−^) SD rats (6–8 weeks old) were injected with Con A (Sigma-Aldrich) at 30 mg/kg body weight by the tail vein. The *Eogt*^+/+^ and *Eogt*^−/−^ groups each had 60 healthy adult male SD rats, which were randomly divided into 5 groups. Con A treatment was used to trigger AIH in a few hours, leading to hepatic dysfunction within 24 h. At 0, 12, 24, 48, and 72 h after injection, the experimental rats were sacrificed, and peripheral blood, and liver, spleen, and thymus samples were collected for further analysis.

### Liver function marker analysis

Serum alanine aminotransferase (ALT) and aspartate aminotransferase (AST) levels in *Eogt*^+/+^ and *Eogt*^−/−^ SD rats were detected on an Olympus AU 2700 analyzer (Olympus, Tokyo, Japan) according to the manufacturer's instructions.

### Flow cytometry analysis of T cell subpopulations in tissues

Over the last few decades, Tregs have been demonstrated to prevent CD4^+^ T lymphocyte-mediated inflammatory diseases, including AIH (25). To assess whether Tregs are regulated by *Eogt* deletion, the population of CD4^+^FoxP3^+^ cells (Tregs) was detected in the liver of *Eogt*^+/+^ and *Eogt*^−/−^ rats after Con A injection by flow cytometry. For cell collection from the spleen, liver, and thymus, the organs were mechanically disrupted in a coffee grinder. Then, spleen and thymus cell suspensions were passed through a fine, 50-μm nylon mesh, and cells were collected by centrifugation at 300 × *g* for 5 min. Liver cell suspension was centrifuged at 50 × *g* for 3 min twice to eliminate hepatocytes; non-parenchymal cells in the liver were collected following Percoll gradient separation. Cells were collected from peripheral blood by centrifugation at 2,000 × *g* for 15 min. Erythrocytes were removed by treating splenic cells with red blood cell lysis buffer (0.15 M NH_4_Cl, 1.0 mM KHCO_3_, 0.1 mM EDTA, pH 7.2) for 5 min and washing twice with cold phosphate-buffered saline (PBS). The resulting cells (1 × 10^6^) were resuspended in PBS and incubated with the following anti-rat antibodies for 30 min at 4°C: anti-CD3-PerCP (eBioscience), anti-CD4-FITC (eBioscience), anti-CD25-APC (BD Bioscience), anti-Foxp3-PE (BD Bioscience), and isotype control antibodies (eBioscience, San Diego, CA). After incubation, the cells were washed twice with fluorescence-activating cell sorter (FACS) washing buffer, and 4 × 10^5^ cells were collected by FACS Vantage SE (FACSCalibur, Becton Dickinson, San Jose, CA, USA) and analyzed with the CellQuest software (CellQuest Pro, Becton Dickinson).

### CD4^+^ T cell isolation and Con A treatment

Con A-induced AIH is characterized by CD4^+^ T lymphocyte infiltration in the liver, and CD4^+^ T lymphocyte-mediated immune responses play an important role in the development and progression of AIH (26). Therefore, to assess whether loss of *Eogt* enhances CD4^+^ T lymphocyte infiltration to further aggravate Con A-induced hepatic dysfunction, we monitored the dynamic changes of the CD4^+^ T lymphocyte population in the liver. CD4^+^ T cells were positively selected from rat splenocytes on a magnetic-activated cell-sorting system (MACS; Miltenyi Biotec, Germany). High-purity CD4^+^ T cells were seeded in 96-well culture plates (5 × 10^5^ cells/well) and cultured in RPMI 1640 (Gibco, Grand Island, NY, USA) with 10% fetal bovine serum, followed by the addition of 5 μg/mL Con A in 6 well plates. The experiment was repeated at least three times. The same concentration of PBS was added to control wells. All cells were collected after 0, 12, 24, 48, and 72 h for FACS analysis.

### *In vitro* treg differentiation and proliferation assays

T cell subsets were obtained from single-cell suspensions of the spleen were prepared from 6-week-old r*Eogt*^+/+^ or r*Eogt*^−/−^ SD rats. Briefly, the harvested spleens were gently processed by gentle extrusion through a metal mesh into cold PBS, and spleen lymphocytes were isolated from the Percoll interphase. The harvested cells were aliquoted and stained with different antibody combinations for sorting naïve CD4^+^ T cells (anti-CD4-PE, clone W3/25, BioLegend; anti-CD45RC-FITC, clone OX-22, Thermo Fisher Scientific) or Treg cells (anti-CD4-PE and anti-CD25-FITC), and subjected to flow cytometric sorting.

In Treg differentiation assays, the collected naïve CD4^+^T cells (CD4^+^CD45RC^+^) were resuspended in complete RPMI 1640 medium supplemented with 2 ng/ml Rat TGFβ1 (Sino Biological), 100 U/ml Rat IL-2 (R&D systems), 1 μg/ml CD28 mAb (clone JJ319, BioLegend), seeded in 24-well plates pre-coated with anti-CD3ε antibody (clone 1F4, BioLegend), and incubated at 37°C with 5% CO2 for 4 days. Treg differentiation was assessed by Q-PCR analysis of Foxp3 mRNA levels and CD25/Foxp3 staining levels in flow cytometry analysis.

The proliferative capacity of Tregs after rEogt gene knockout was assessed with Cell Counting Kit-8 (CCK-8) according to the manufacturer's instructions. Briefly, the purified Tregs (CD4^+^CD25^+^) were seeded at a density of 5 × 10^3^ cells in 96-well plates in quadruplicate, and incubated for 48 h in complete RPMI 1640 medium with equal amounts of Con A (0, 2, and 4 μg/ml) and recombinant 100 U/ml Rat IL-2 (25 ng/ml, R&D Systems) in a humid atmosphere with 5% CO2 at 37°C. Afterward, the supernatants were pulsed with the CCK-8 solution (1/10), and the cells were incubated for another 2 h at 37°C. Absorbance at 450 nm was determined on a microplate reader (BioTek, MQX200). All experiments were repeated three times.

### Quantitative real-time PCR

Total RNA was isolated with TRIzol reagent (Invitrogen, Carlsbad, CA, USA) according to the manufacturer's instructions. A total of 1 μg RNA was used as a template for single-strand cDNA synthesis using oligo(dT) primers and TransScript®RT/RI Enzyme Mix (TransGen Biotech, Beijing, China). The resulting cDNA was amplified with TransStart® Top Green qPCR SuperMix (TransGen Biotech, Beijing, China) on an ABI Prism 7500 sequence detection system (Applied Biosystems, Foster City, CA, USA), programmed for 94°C for 30 s, followed by 40 cycles of 94°C for 5 s and 60°C for 30 s. Amplification results were analyzed with the ABI Prism 7500 software (Applied Biosystems), and expression levels of the genes of interest were normalized to the corresponding *Gapdh* results. The primer sequences are presented in Table 1.

**Table 1:** Primer sequence used in Q-PCR.

**Gene**		**Primer sequence**
rRBPj	Forward	GGGTGTAGCCTCCTTTCT
	Reverse	TGTATTTGGACGATGGTTT
rNotch1	Forward	ATGACTGCCCAGGAAACAAC
	Reverse	ATGACTGCCCAGGAAACAAC
rJagged1	Forward	GCTTCGGCTCAGGGTCTA
	Reverse	AGTCACCTGGGAGTTTGC
rJagged2	Forward	CCAGGAAGTGGTCATATTCACGA
	Reverse	GCAGACAAGGCTTCCAACCAC
rHes1	Forward	GCTTCAGCGAGTGCATGAAC
	Reverse	CGGTGTTAACGCCCTCACA
rFoxp3	Forward	GGCCCTTCTCCAGGACAGA
	Reverse	GCTGATCATGGCTGGGTTGT
rCTLA-4	Forward	GGACTGAGGGCTGCTGACAC
	Reverse	GGCATGGTTCTGGATCGATG
rGITR	Forward	GCAGACTTTGGACCAACTGTTC
	Reverse	AGCGGCTGG GTATTGACCT
rGapdh	Forward	5′-AGGTCGGTGTGAACGGATTTG-3′
	Reverse	3′-GGGGTCGTTGATGGCAACA-5′

### Western blot

The samples were lysed in a buffer containing 50 mM Tris-HCl (pH 7.4), 150 mM NaCl, 1% NP40, 0.25% Na-deoxycholate, and 1 mM phenylmethylsulfonyl fluoride. After centrifugation, protein samples were subjected to 10% sodium dodecyl sulfate-polyacrylamide gel electrophoresis and transferred onto polyvinylidene fluoride (PVDF) membranes (Roche, Mannheim, Germany). The membranes were then blocked in TBST (1 mM Tris-HCl, pH 7.4, 150 mM NaCl, 0.05% Tween-20) containing 5% skim milk for 60 min, and incubated overnight at 4°C with diluted primary antibodies against GAPDH, β-actin, NALP2 and EOGT (Abcam, Database link: Q5NDL2), as well as cleaved caspase 3, cleaved caspase 7 and RBPJ (Cell Signaling Technology). After 3 washes with TBST for 5 min, the membranes were incubated with secondary antibodies in blocking buffer at room temperature for 1 h. Finally, chemiluminescence was used for detection, and images were acquired by autoradiography.

## Immunoprecipitation

For immunoprecipitation of total Notch1, lymphocyte lysates were incubated with Notch1 antibody (clone mN1A, Santa Cruz Biotechnology) at 2 mg/ml for 2 h at 4°C, and further incubated with protein G-coupled Sepharose beads (20 μL) for 1 h. After 5 washes with ice-cold washing buffer, total protein was eluted with SDS-PAGE sample loading buffer, and separated by 7% SDS-polyacrylamide gel electrophoresis followed by transfer onto polyvinylidene difluoride membranes (Millipore, Burlington, MA, USA). Finally, immunoblotting for the detection of O-GlcNAc and Notch1 was performed using anti-O-GlcNAc (clone CTD110.6, BioLegend) and anti-NOTCH1 antibodies, respectively.

### Luminex multiplex assays

A Luminex assay (Luminex®) was used to determine the serum levels of 20 cytokines, including several interleukins (ILs), chemokines, and cytokines: GM-CSF, IFN-γ, IL-1β, IL-2, IL-4, IL-5, IL-6, IL-10, IL-13, TNF-a, G-CSF, Eotaxin, GRO-a, IP-10, MCP-1, MCP-3, MIP-1a, MIP-2, RANTES, and TGF-β.

### Statistical analysis

Data are mean ± SEM, and were assessed by Student's *t*-test with the GraphPad Prism 5.01 software (San Diego, CA, USA). *P* < 0.05 was considered statistically significant.

## Results

### *Eogt* knockout rats produced by TALEN-mediated gene inactivation

To generate *Eogt* knockout rats, we designed a TALEN that targets the rat *Eogt* gene in exon 5 (Figure 1A). After transfer of TALEN mRNA-injected fertilized eggs to the uterus of a recipient, eight healthy offspring were produced. PCR products of around 500 bp were amplified from each animal corresponding to the portion of the *Eogt* locus overlapping with the TALEN spacer (Figure 1B). DNA sequence analysis further revealed that the PCR products of two females had a 10-bp deletion (−10 bp) in the spacer region (Figure 1C).

To generate offspring homozygous for this deletion, these female founder rats were mated with wild-type SD males, and six of the F1 offspring harbored the −10 bp allele. Heterozygous F1 offspring were then interbred to produce an F2 offspring, and DNA sequence analysis indicated that four of the F2 offspring were homozygous for the −10 bp allele. Western blot analysis further confirmed the successful establishment of *Eogt* knockout rats (*Eogt*^−/−^) by the TALEN method, since the EOGT protein was detected in various tissues (heart, liver, spleen, lung, kidney, thymus and lymph nodes) of wild-type rats but not in any of the −10 bp animals (Figure 1D). Furthermore, body weights in *Eogt*^−/−^ rats were significantly lower compared with those of *Eogt*^+/+^ animals on day 20 after birth (Figure 1E).

### Con A-induced liver injury is aggravated in *Eogt^−/−^* rats

**Figure 2:**
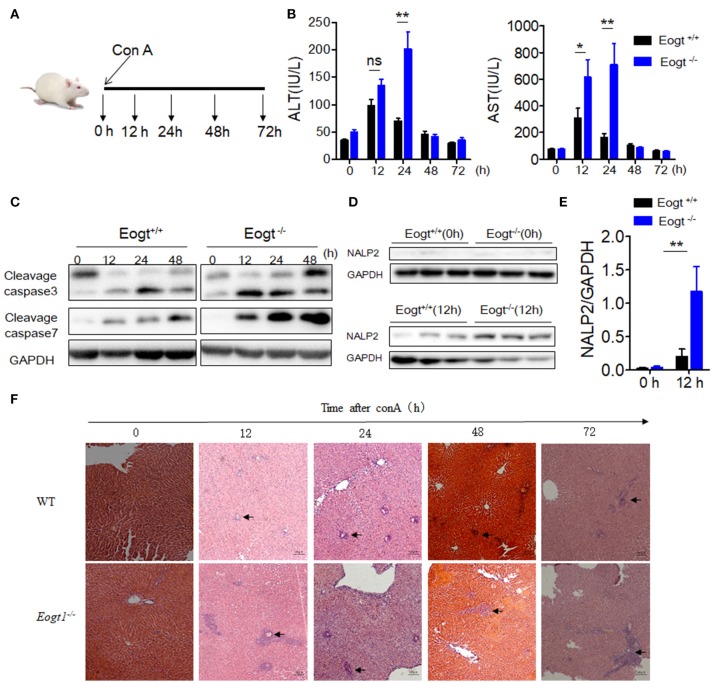
Knockout Eogt aggravates concanavalin-A induced liver injury. **(A)** Schematic outline of concanavalin-A (Con A) administration in Eogt^+/+^ and Eogt^−/−^ rats. **(B)** ALT and AST levels in Eogt^+/+^ and Eogt^−/−^ rats after Con-A injection at 0, 12, 24, 48, and 72 h; **(C)** Western blot analysis of cleaved caspases 3 and 7 in the liver of Eogt+/+ and Eogt^−/−^ rats after Con-A injection at 0, 12, 24, and 48 h; **(D)** Western blot analysis of NALP2 protein levels in the liver of Eogt^+/+^ and Eogt^−/−^ rats after Con-A injection at 0 and 12 h; **(E)** Quantification of NALP2 protein expression in **(D)**. **(F)** Representative H and E staining of liver sections after Con-A injection at 0, 12, 24, and 48 h. ^*^p < 0.05; ^**^p < 0.01, vs. WT control group.

After Con A injection, both *Eogt*^+/+^ and *Eogt*^−/−^ rats displayed hepatic dysfunction as evidenced by dynamic changes of ALT and AST levels (Figure 2A). At 12 h after Con A injection, serum ALT and AST levels were markedly increased in both groups; however, these levels were much higher in *Eogt*^−/−^ rats. ALT and AST levels decreased from 12 to 24 h in *Eogt*^+/+^ rats, but remained high in *Eogt*^−/−^ animals. From 24 to 72 h, ALT and AST levels were gradually restored to normal levels in both groups (Figure 2B).

Western blot analysis further showed that the levels of apoptotic marker proteins, including cleaved caspases 3 and 7, were increased in the liver of Con A-treated *Eogt*^+/+^ rats (Figure 2C), with more pronounced increases observed in *Eogt*^−/−^ rats, indicating that *Eogt* knockout aggravated hepatic dysfunction (Figure 2C). Moreover, Con A-induced liver dysfunction and apoptosis were accompanied with overtly increased NALP2 levels, indicating the activation of inflammatory responses in the liver; the alteration was 3-fold higher in *Eogt*^−/−^ rats compared with *Eogt*^+/+^ animals (Figures 2D,E). Histological analysis confirmed that Con A-induced acute liver injury was more severe in *Eogt*^−/−^ rats (Figure 2F).

### Con A-induced *Eogt^−/−^* rats show abnormal treg activation

**Figure 3:**
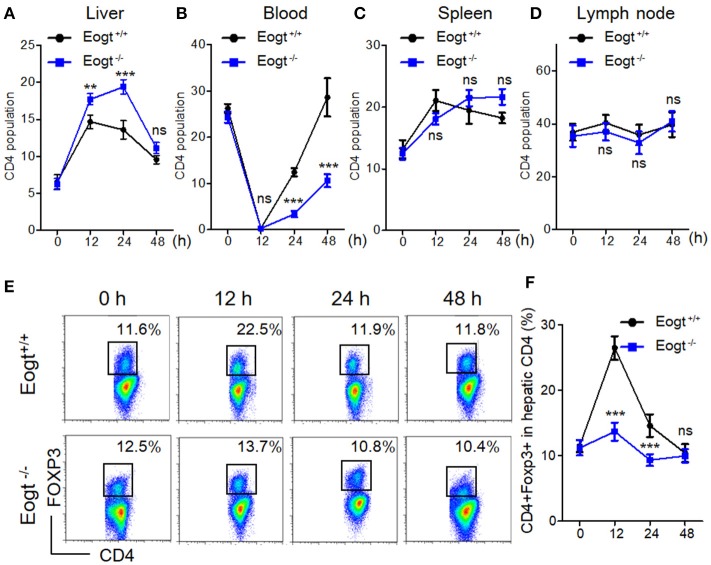
Abnormal activation of Tregs in Eogt^−/−^ rats after Concanavalin-A treatment. **(A–D)**. Flow cytometry analysis of CD4^+^ T cells in the Liver **(A)**, Blood **(B)**, Spleen **(C)** and Lymph nodes **(D)** of Eogt^+/+^ and Eogt^−/−^ rats after Con-A injection at 0, 12, 24, and 48 h; **(E)** Flow cytometry assessment of CD4^+^FOXP3^+^ (Treg) cells in the Liver of Eogt^+/+^ and Eogt^−/−^ rats after Con-A injection at 0, 12, 24, and 48 h; **(F)** Quantification of Tregs in **(E)**. ^**^p < 0.01; ^***^p < 0.001, vs. WT control group.

In Con A-treated *Eogt*^+/+^ rats, the percentage of liver CD4^+^ T lymphocyte was obviously increased in the first 12 h, and gradually decreased thereafter (Figure 3A). By contrast, Con A-treated *Eogt*^−/−^ rats showed a continuous increase in liver CD4^+^ T lymphocytes over the first 24 h, which then declined to the normal level (Figure 3A). The frequencies of CD4^+^ T lymphocytes in peripheral blood were significantly decreased at 1 h after Con A injection in both *Eogt*^+/+^ and *Eogt*^−/−^ rats (Figure 3B), with no difference between the two groups. However, the recovery rate of peripheral blood CD4^+^ T lymphocytes over the following 12–36 h was higher in *Eogt*^+/+^ rats (Figure 3B). There were no significant differences in the dynamic changes of CD4^+^ T lymphocytes in the spleen and lymph nodes between the two groups (Figures 3C,D).

In line with a previous report (26), we found that the population of liver CD4^+^Foxp3^+^ cells (Treg) was increased from 11.48 ± 0.93% to 26.42 ± 1.80% 12 h after Con A treatment in *Eogt*^+/+^ rats, and returned to baseline levels at 24 and 48 h (Figures 3E,F). However, in *Eogt*^−/−^ rats, the proportion of Tregs only increased slightly from 11.18 ± 1.15% to 13.67 ± 1.39% at 12 h after Con A treatment, which was significantly lower compared with that of *Eogt*^+/+^ animals (Figures 3E,F).

### EOGT knockout inhibits the generation of itregs from CD4^+^CD45RC^+^ T cells *in vitro*

**Figure 4:**
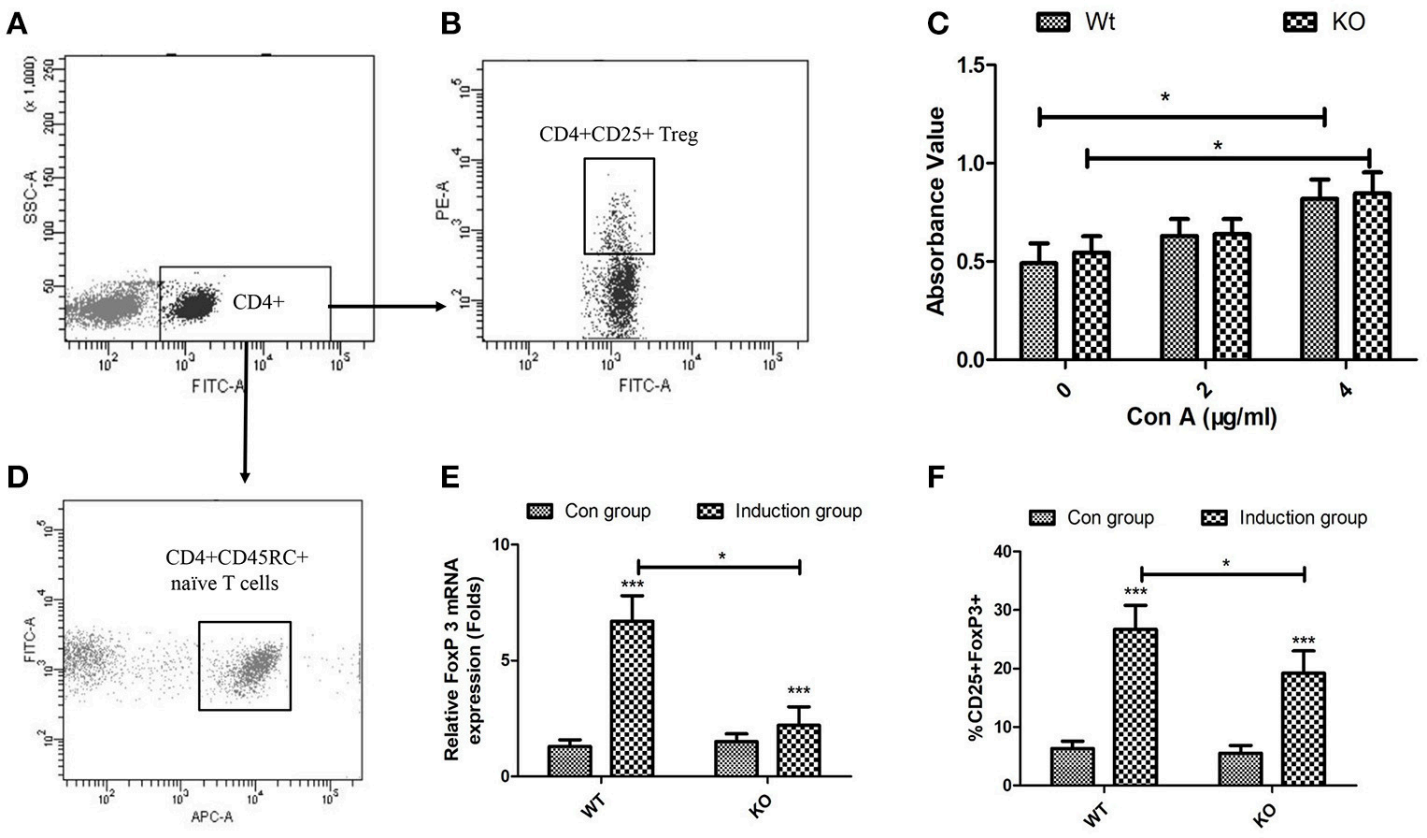
Effects of EOGT knockout on Treg cell proliferation and differentiation. Flow cytometry for sorting naive CD4^+^ T cells (CD4^+^CD45RC^+^) **(A,D**) and CD4^+^CD25^+^ Tregs **(A,B**) from rat spleens; **(C)** CD4^+^CD25^+^ Tregs from Eogt^+/+^ and Eogt^−/−^ rats treated with IL-2 without or with different Con A concentrations for 48 h; **(E)** Foxp3 mRNA levels and **(F)** percentages of regulatory T cells (CD25^+^Foxp3^+^) differentiated from naive CD4^+^ T cells (CD4^+^CD45RC^+^). Data are mean ± SD from three independent experiments. ^*^P < 0.05; ^***^p < 0.001, vs. WT control group.

To directly assess the role of EOGT on Treg proliferation and differentiation, we adopted flow cytometry to sort naive CD4^+^ T cells (CD4^+^CD45RC^+^) (Figures 4A,D) and CD4^+^CD25^+^ Tregs (Figures 4A,B) from rat spleens. Although Con A could promote Treg proliferation in the presence of IL-2, no statistically significant differences were observed between the WT and KO groups with or without Con A (Figure 4C). Next, we adopted an *in vitro* culture system where naive CD4^+^ T cells (CD4^+^CD45RC^+^) were differentiated into FoxP3^+^ Tregs upon anti-CD3 mAb stimulation in the presence of TGF-β1, rIL-2 and anti-CD28 mAb. However, Foxp3 mRNA levels and the percentage of inducible regulatory T cells (CD25+Foxp3+) in KO induction groups were significantly lower compared with those of WT induction groups (Figures 4E,F).

### Eogt suppression alters the serum levels of chemokines

**Figure 5:**
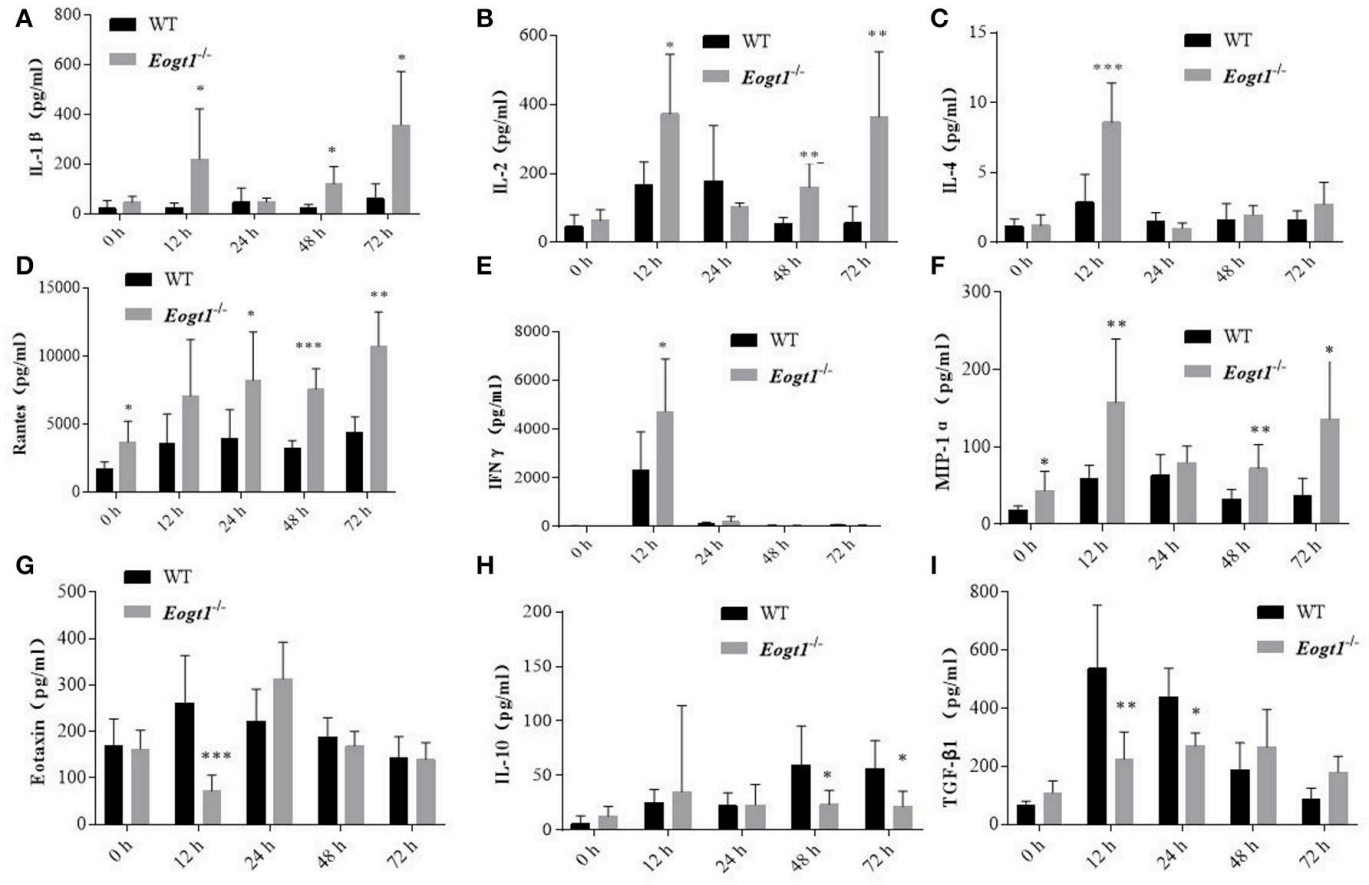
Luminex multiplex analysis of cytokine levels in rat serum. Column charts respectively show serum levels of 9 cytokines in Eogt^+/+^ and Eogt^−/−^ rats after Con-A injection at 0, 12, 24, 48, and 72 h. Data are mean ± SD from three independent experiments. ^*^P < 0.05; ^**^P < 0.01, vs. WT control group. **(A)** IL-1β, **(B)** IL-2, **(C)** IL-4, **(D)** Rentes, **(E)** IFN-r, **(F)** MIP-1α, **(G)** Extaxin, **(H)** IL-10, **(I)** TGF-β1.

Since many cytokines and chemokines play important roles in the development of autoimmune hepatitis diseases, we next determined the serum levels of select cytokines, including interleukins (ILs) and other inflammatory cytokines. Before Con A injection, serum RANTES and MIP-1α levels in *Eogt*^−/−^ rats were significantly higher than those of *Eogt*^+/+^ animals (*p* < 0.05, Figures 5D,F). With autoimmune hepatitis associated with Con A, the serum levels of some inflammatory cytokines (IL-2/4/10, IFNγ and TGF-β) were altered to varying degrees. However, as shown in Figure 5, serum IL-1β (12, 48, and 72 h), IL-2 (12, 48, and 72 h), IL-4 (12 h), RANTES (24, 48, and 72 h), IFNγ (12 h) and MIP-1α (12, 48, and 72 h) levels in Con A-treated *Eogt*^−/−^ rats were significantly higher compared with those of Con A-treated *Eogt*^+/+^ animals, while serum Eotaxin (12 h), IL-10 (48 and 72 h) and TGF-β (12 and 24 h) were significantly lower (*p* < 0.05 or 0.01). No variations of other cytokines in serum were observed (data not shown).

### EOGT inhibits treg activation via the notch signaling pathway

**Figure 6:**
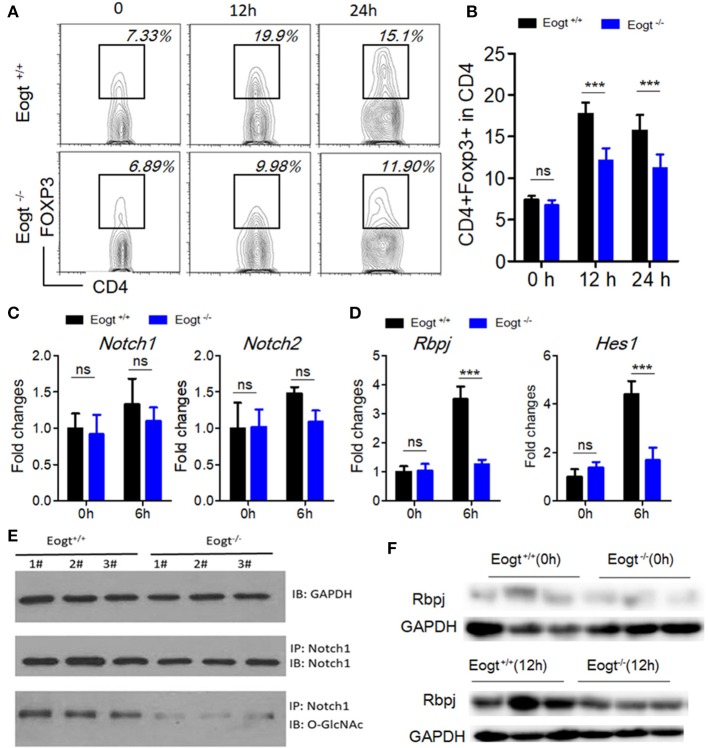
Knockout of Eogt inhibits Treg differentiation via the NOTCH signaling pathway. **(A)** Flow cytometry analysis of CD4^+^FOXP3^+^ (Treg) cells in Eogt^+/+^ (*n* = 6) and Eogt^−/−^ (*n* = 6) CD4^+^ T cells after *in vitro* treatment with Con A; **(B)** Quantification of Tregs in **(A)**; **(C)** Quantitative PCR analysis of *Nocth1* and *Nocth2* in Eogt^+/+^ (*n* = 6) and Eogt^−/−^ (*n* = 6) CD4^+^ T cells after *in vitro* treatment with Con A; **(D)** Quantitative PCR analysis of *Rbpj* and Hes1 in Eogt^+/+^ (*n* = 6) and Eogt^−/−^ (*n* = 6) CD4^+^ T cells after *in vitro* treatment with Con A; Western blot analysis of O-GlcNAc and Notch1 levels in lymphocytes **(E)** and Rbpj expression amounts in Eogt^+/+^ (*n* = 6) and Eogt^−/−^ (*n* = 6) CD4^+^ T cells after *in vitro* treatment with Con A **(F)**. ^***^p < 0.001, vs. WT control group.

To further assess the relationship between EOGT-mediated glycosylation and Treg activation, we performed MACS to separate CD4^+^ T cells from *Eogt*^+/+^ and *Eogt*^−/−^ rat spleens. Flow cytometry analysis confirmed that a purity for the isolated CD4^+^ T cells of 96.43 ± 2.67%. Next, we cultured the isolated CD4^+^ T cells under Con A stimulation (5 μg/mL), and detected the Treg population by FACS. After 12 h, the proportion of Tregs were increased from 7.37 ± 0.47% to 17.73 ± 1.35% in the *Eogt*^+/+^ CD4^+^ T population (Figures 6A,B), whereas this increase was significantly lower in the *Eogt*^−/−^ CD4+ T cells, from 6.73 ± 0.62% to 12.10 ± 1.50% (Figures 6A,B). Notch signaling has been reported to promote the differentiation and survival of Tregs (27–29). Meanwhile, glycosylation of the extracellular domain of Notch is important for activating Notch signaling (30). To determine whether loss of *Eogt* suppresses Notch signaling to prevent Treg differentiation, cultured CD4^+^ T cells were collected after 6 h of Con A stimulation for gene expression analysis. Con A treatment did not alter the expression levels of *Notch1* and *Notch2* in *Eogt*^+/+^ and *Eogt*^−/−^ CD4^+^ T cells (Figure 6C); meanwhile, Eogt knockout decreased Notch1 protein O-GlcNAcylation levels in lymphocytes in *Eogt*^−/−^ rats (Figure 6E). However, the expression levels of *Rbpj* and *Hes1*, Notch signaling-activated genes, were markedly induced in *Eogt*^+/+^ CD4^+^ T cells but not in *Eogt*^−/−^ counterparts (Figure 6D). Furthermore, Western blot demonstrated that *Eogt* knockout reduced the activation of RBPJ, the major transcriptional effector of Notch signaling (Figure 6F).

## Discussion

PTMs of proteins constitute one of the most effective methods to dynamically and rapidly regulate protein functions, and play important roles in several physiological and pathological processes; indeed, abnormalities of protein PTMs are both the causes and consequences of various diseases (31). In this study, the TALEN technology was applied to knock out the rat *Eogt* gene, which encodes the enzyme that catalyzes protein glycosylation, and demonstrated that this glycosylation deficiency could prevent Treg differentiation by suppressing Notch signaling, leading to CD4^+^ T lymphocyte infiltration that resulted in the aggravation of hepatic dysfunction in Con A-induced AIH.

AIH is the most typical autoimmune disease, and characterized by a T-cell-rich infiltrate (32). In the course of disease development, effector T cells from peripheral blood are recruited to the inflamed liver, leading to the apoptosis of hepatocytes. Then, activated Tregs suppress the proliferation and cytokine secretion of infiltrating effector T cells (33). Previous studies demonstrated a reduction of Treg frequency in peripheral blood from AIH patients (10, 11), suggesting that Tregs are recruited to the inflamed liver along with effector T cells to control inflammation (12). In line with these previous reports, this study demonstrated a rapid reduction of CD4^+^ T cells in peripheral blood and an accumulation of CD4^+^ T cells in the liver following Con A treatment; however, the frequency of Tregs in the liver was largely different between *Eogt*^+/+^ and *Eogt*^−/−^ rats, with only a slight increase in the latter and a marked increase in the former. This *in vivo* finding might indicate that the recruited Tregs from peripheral blood only represent a small population of liver Tregs, and the main liver Treg population might instead originate from T cell differentiation in response to the hepatic microenvironment (34). We sorted Tregs (CD4^+^CD25^+^FoxP3^+^) from liver samples after Con A-induced autoimmune liver injury at 12 h, and compared the mRNA levels of CTLA-4 and GITR in CD4^+^CD25^+^Foxp3^+^ (Treg) cells. As shown in Figure S1, there were no significant differences in mRNA expression levels of CTLA-4 and GITR between the two subsets of WT and KO rats, suggesting that abnormal Treg activation caused by Eogt knockout could be independent of Tregs' functional makers.

*In vitro* and *in vivo* studies have demonstrated that T cell receptor signaling, cytokines, and other signaling pathways play important roles in the development and differentiation of Tregs in disease (35–37). In a liver with AIH, the presence of transforming growth factor-beta released from hepatocytes or other activated non-parenchymal cells could promote Treg differentiation (38, 39). We further showed that Con A also regulated the differentiation of Tregs from CD4^+^ T lymphocytes *in vitro*. Previous studies have shown that Con A-treated CD4^+^ T cells have significantly increased levels of Notch1 (40), and activation of the Notch signaling pathway has been detected in induced Tregs (41). Although Con A did not upregulate *Notch1* in the CD4^+^ T cell population in the present study, Notch signaling was activated upon Con A stimulation, which promoted the differentiation of Tregs mediated by glycosylation. Protein structure analysis revealed that the extracellular domain of Notch contains epidermal growth factor-like repeats, representing a consensus site for O-glycosylation catalyzed by EOGT (42). In addition, EOGT-mediated Notch signaling pathway activation has been confirmed in *Eogt*^−/−^ mice (42).

The current study had several limitations. First, due to the lack of well-established *in vitro* rat Treg cell-differentiation conditions, we could only refer to the reported method of *in vitro* mouse Treg differentiation from naïve T cells. Secondly, as many immune cells as possible should be assessed in other immune organs (bone marrow, peripheral blood, spleen and lymph nodes etc.) to explore the role of Eogt knockout in the immune system. Thirdly, because liver samples in this study were not properly preserved, we could not detect the CD69 activation marker and the cleaved Notch intracellular domain (NICD) on CD4^+^ T cells.

In summary, this study demonstrated that EOGT plays a critical role in AIH by regulating Treg differentiation via Notch signaling. In EOGT-deficient rats, Treg differentiation was clearly impaired due to inactivated Notch signaling, resulting in abnormal infiltration of the T cell population into the liver, which aggravates hepatic injury. Therefore, this study revealed a regulatory role for glycosylation in the pathogenesis of AIH, highlighting a potential therapeutic target.

## Ethics statement

This study was carried out in accordance with the recommendations of the international guidelines for the care and use of laboratory animals, Experimental Animal Care and Use Committee of Peking University Health Science Center. The protocol was approved by the Experimental Animal Care and Use Committee of Peking University Health Science Center.

## Author contributions

HW, XH, and YL contributed to research design. XH, YL, JW, JM, SZ, XY, LH, JY, and MG, performed the research. FX and SZ provided innovative views and opinions. XH and YL analyzed the data. XH and SZ wrote the manuscript. HW revised the manuscript.

### Conflict of interest statement

The authors declare that the research was conducted in the absence of any commercial or financial relationships that could be construed as a potential conflict of interest.
