# Supplementary figures and images for: Deficient O-GlcNAc Glycosylation Impairs Regulatory T Cell Differentiation and Notch Signaling in Autoimmune Hepatitis
Source: Front Immunol. 2018 Oct 9;9:2089. doi: 10.3389/fimmu.2018.02089 (PMC6189470; doi:10.3389/fimmu.2018.02089)

Figure S1

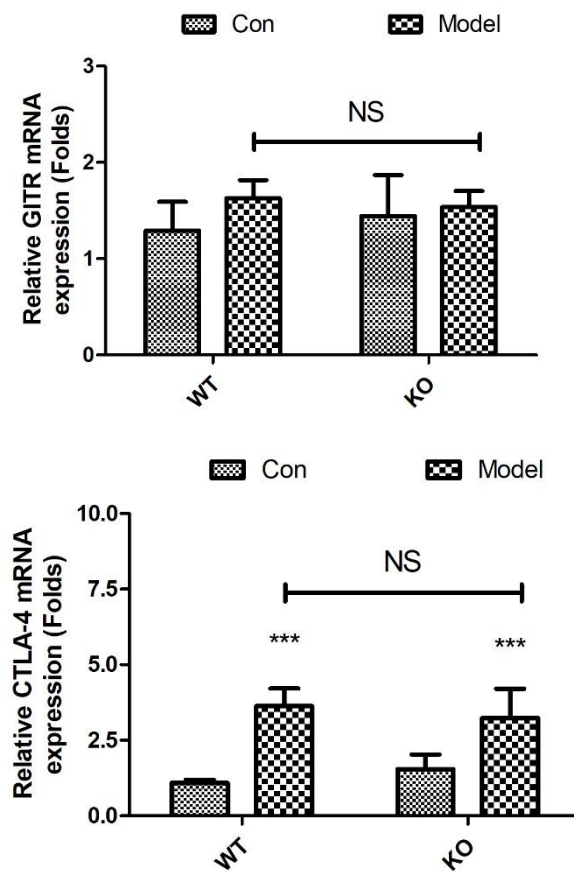

Supplement: Supplementary file 1 [file Data_Sheet_1.PDF]
